# Purification of Sewage

**Published:** 1907-02

**Authors:** 


### PURIFICATION OF SEWAGE.

---

A valuable contribution to the literature on the disposal and purification of sewage has just been issued by the United States Geological Survey as Water-Supply and Irrigation Paper No. 185. Investigations no the purification of Boston sewage with a history of the sewage-disposal problem, by C. E. A. Winslow and E. B. Phelps. The volume of sewage discharged by modern communities is so large and the character of all kinds of sewage is always so objectionable that the so-called sewage-disposal problem becomes, from the economic as well as the sanitary point of view, one of the most serious with which American cities have to deal. It is of vital importance to every community to secure such a disposal of obnoxious sewage as will avoid the creation of any unsanitary focus or foci in the environment or any infringement of the laws of hygiene and sanitation.

The investigations described in this publication were made at the Sanitary Research Laboratory and Sewage Experiment Station of the Massachussetts Institute of Technology under the direction of Prof. William T. Sedgwick. The station at which the work was carried on is situated on the line of the main trunk sewer of the south Metropolitan district of Boston at a poin where it contains the sewage of about half a million people. At this station pumps were installed and tanks were constructed for tests of the various methods of sewage purification. The results of this work and the practical conclusions that have been drawn are given in Water Supply Paper No. 185, which may be obtained on application to the Director of the United States Geological Survey, Washington, D. C. These results are by no means applicable merely to yarge cities, but contain lessons of practical value to all communities having to deal with the ever present sewage-disposal problem. The description of the experiments is preceded by a careful and elaborate historical review of the whole sewage-disposal problem from its origin in the wide adoption of the water-carriage system up to the present time, when that system has become practically universal. This interesting review cannot fail to be of the highest value to expert engineers, sewage commissioners, and cities all over the United States, especially to those numerous small communities that are confronted, perhaps for the first time, with a problem that means so much for the health as well as the finances of the citizens.

